# Multimorbidity and overall survival among women with breast cancer: results from the South African Breast Cancer and HIV Outcomes Study

**DOI:** 10.1186/s13058-023-01603-w

**Published:** 2023-01-23

**Authors:** Oluwatosin A. Ayeni, Maureen Joffe, Witness Mapanga, Wenlong Carl Chen, Daniel S. O’Neil, Boitumelo Phakathi, Sarah Nietz, Ines Buccimazza, Sharon Čačala, Laura W. Stopforth, Judith S. Jacobson, Katherine D. Crew, Alfred I. Neugut, Duvern Ramiah, Paul Ruff, Herbert Cubasch, Tobias Chirwa, Valerie McCormack, Lisa K. Micklesfield, Shane A. Norris

**Affiliations:** 1 Strengthening Oncology Services Research Unit, Faculty of Health Sciences, University of the Witwatersrand, Johannesburg, South Africa; 2 Department of Radiation Oncology, Faculty of Health Sciences, University of the Witwatersrand, Johannesburg, South Africa; 3 SAMRC/Wits Developmental Pathways for Health Research Unit, Department of Paediatrics, Faculty of Health Sciences, University of the Witwatersrand, Johannesburg, South Africa; 4 National Cancer Registry, National Health Laboratory Service, Johannesburg, South Africa; 5 Sydney Brenner Institute for Molecular Bioscience, Faculty of Health Sciences, University of the Witwatersrand, Johannesburg, South Africa; 6 Sylvester Comprehensive Cancer Center and Department of Medicine, Miller School of Medicine, University of Miami, Miami, FL USA; 7 Department of Surgery, School of Clinical Medicine, University of KwaZulu-Natal, KwaZulu-Natal, South Africa; 8 Department of Surgery, Faculty of Health Sciences, University of the Witwatersrand, Johannesburg, South Africa; 9 Department of Surgery, Ngwelezana Hospital, Empangeni and University of KwaZulu-Natal, Empangeni, KwaZulu-Natal South Africa; 10 Departments of Surgery and Radiation Oncology, Grey’s Hospital, University of KwaZulu-Natal, Pietermaritzburg, KwaZulu-Natal South Africa; 11 Herbert Irving Comprehensive Cancer Center, Vagelos College of Physicians and Surgeons, Columbia University, New York, NY USA; 12 Department of Epidemiology, Mailman School of Public Health, Columbia University, New York, NY USA; 13 Department of Medicine, Vagelos College of Physicians and Surgeons, Columbia University, New York, NY USA; 14 Division of Medical Oncology, Department of Internal Medicine, Faculty of Health Sciences, University of the Witwatersrand, Johannesburg, South Africa; 15 School of Public Health, Faculty of Health Sciences, University of the Witwatersrand, 27 St Andrews Road, Parktown, Johannesburg, 2193 South Africa; 16 Environment and Lifestyle Epidemiology Branch, International Agency for Research on Cancer, (IARC/WHO), Lyon, France; 17 Global Health Research Institute, School of Human Development and Health, University of Southampton, Southampton, UK

**Keywords:** Breast cancer, Multimorbidity, Chronic conditions, Survival, South Africa

## Abstract

**Background:**

Breast cancer survival in South Africa is low, but when diagnosed with breast cancer, many women in South Africa also have other chronic conditions. We investigated the impact of multimorbidity (≥ 2 other chronic conditions) on overall survival among women with breast cancer in South Africa.

**Methods:**

Between 1 July 2015 and 31 December 2019, we enrolled women newly diagnosed with breast cancer at six public hospitals participating in the South African Breast Cancer and HIV Outcomes (SABCHO) Study. We examined seven chronic conditions (obesity, hypertension, diabetes, HIV, cerebrovascular diseases (CVD), asthma/chronic obstructive pulmonary disease, and tuberculosis), and we compared socio-demographic, clinical, and treatment factors between patients with and without each condition, and with and without multimorbidity. We investigated the association of multimorbidity with overall survival using multivariable Cox proportional hazard models.

**Results:**

Of 3,261 women included in the analysis, 45% had multimorbidity; obesity (53%), hypertension (41%), HIV (22%), and diabetes (13%) were the most common individual conditions. Women with multimorbidity had poorer overall survival at 3 years than women without multimorbidity in both the full cohort (60.8% vs. 64.3%, *p* = 0.036) and stage groups: stages I–II, 80.7% vs. 86.3% (*p* = 0.005), and stage III, 53.0% vs. 59.4% (*p* = 0.024). In an adjusted model, women with diabetes (hazard ratio (HR) = 1.20, 95% confidence interval (CI) = 1.03–1.41), CVD (HR = 1.43, 95% CI = 1.17–1.76), HIV (HR = 1.21, 95% CI = 1.06–1.38), obesity + HIV (HR = 1.24 95% CI = 1.04–1.48), and multimorbidity (HR = 1.26, 95% CI = 1.13–1.40) had poorer overall survival than women without these conditions.

**Conclusions:**

Irrespective of the stage, multimorbidity at breast cancer diagnosis was an important prognostic factor for survival in our SABCHO cohort. The high prevalence of multimorbidity in our cohort calls for more comprehensive care to improve outcomes for South African women with breast cancer.

**Supplementary Information:**

The online version contains supplementary material available at 10.1186/s13058-023-01603-w.

## Background

Breast cancer remains the most commonly diagnosed cancer and the leading cause of cancer death among women worldwide [1]. It is also the most common cancer among women in South Africa accounting for 22.6% of all female cancers and 16% of cancer deaths amongst women [2]. In high-income countries (HICs), which have population-based mammography screening, early-stage diagnosis, and access to diagnostic and treatment resources, breast cancer has a relatively favourable prognosis; in the USA, the 5-year survival is 80% among African American women and 91% among non-Hispanic White women [3]. Among black South African women, 3-year overall survival is estimated to be 59% [4] mainly due to late-stage diagnosis and sub-optimal multi-modality treatment access and completion.

The increasing prevalence of chronic health conditions, and consequent increasing prevalence of multimorbidity defined as the presence of two or more chronic conditions in one person [5], is a major challenge facing healthcare systems, including those in South Africa [6]. Patients from socio-economically disadvantaged communities face numerous complex social and medical conditions that combine syndemically to affect the quality of life and survival outcomes adversely [7, 8]. This clustering of social and health problems is often overlooked in models of epidemiological transition investigations. As infectious disease mortality declines and populations age, more individuals are living longer with multimorbidity [9]. They impose an ever-increasing burden on overstretched public health systems and pose complex problems for patient management and clinical treatment, and patients experience reduced quality of life and increased mortality [6, 9]. Lifestyle modification and treatment advances have significantly reduced the mortality rate from multimorbidity in HICs [10], but have not reached many upper-middle-income countries like South Africa on which multimorbidity is now imposing serious health, social, and health system resource burdens [11].

Currently, among women newly diagnosed with breast cancer in South Africa, approximately 81% are estimated to have at least one self-reported chronic health condition, and 44% multimorbidity [12]. Although many prior studies have assessed the association between individual chronic conditions and survival in patients with breast cancer, few have quantified the multimorbidity burden, in particular the associations of multiple simultaneous conditions with survival [13]. A review evaluating the impact of multimorbidity on breast cancer guideline adherence for treatment decision-making concluded that multimorbidity can have a profound impact on health, day-to-day function, and mortality among patients with breast cancer [14]. The population-based FOCUS cohort of 3,672 Dutch women with breast cancer aged ≥ 65 years showed that the number of comorbidities was associated with worse overall survival [15].

In our previous studies, we found that women who were obese and hypertensive were more likely to present with early-stage breast cancer and that multimorbidity was not associated with the stage of breast cancer diagnosis [12, 16]. We also found that in early-stage breast cancer, women with multimorbidity were less likely to have surgery, less likely to receive chemotherapy, and more likely to receive endocrine therapy than those with a low multimorbidity burden [12]. We now describe the impact of multimorbidity on overall survival in women with breast cancer enrolled in the South African Breast Cancer and HIV Outcomes (SABCHO) Study.

## Methods

### Study design and settings

The SABCHO Study is a prospective multi-centre cohort study of patients diagnosed with breast cancer at six tertiary hospital sites in the Gauteng and KwaZulu-Natal provinces of South Africa: Charlotte Maxeke Johannesburg Academic Hospital (CMJAH), Johannesburg; Chris Hani Baragwanath Academic Hospital (CHBAH), Johannesburg; Addington Hospital, Durban; Inkosi Albert Luthuli Central Hospital, Durban; Grey’s Hospital, Pietermaritzburg; and Ngwelezana Hospital, Empangeni. Women were enrolled in the breast units of these hospitals. The SABCHO Study was approved by the University of the Witwatersrand Human Research Ethics Committee, the University of KwaZulu-Natal Biomedical Research Committee, and the Institutional Review Board of Columbia University.

## Recruitment of participants

Women who were ≥ 18 years of age, had resided in South Africa for ≥ 5 years, and were newly diagnosed with invasive breast cancer were eligible for enrolment in the SABCHO Study. Women who had a previous cancer diagnosis (other than non-invasive cervical cancer or nonmelanoma skin cancer), or were unable to give informed consent, were excluded. The SABCHO protocol has been published previously [17]. For this analysis, we included women diagnosed between 1 July 2015 and 31 December 2019.

## Data collection and processing

Socio-demographic information (such as age, ethnicity, marital status, the highest level of education, and employment status) and lifestyle factors (alcohol consumption and smoking) were collected at diagnosis. Body weight and height were measured at enrolment. We generated a score for household socio-economic status (HSES) based on self-reported household possessions and facilities (homeownership, car ownership, microwave, washing machine, indoor flush toilet, indoor running water, and gas or electric). One point was allocated to each possession, and we categorized the total HSES score (range 0–7) into low (scores 0–5) and high (scores 6–7). We categorized the hospital site as CHBAH vs. CMJAH vs. Durban vs. Greys vs. Ngwelezana. The two hospitals in Durban share facilities and staff and are analysed here as a single unit. Participants were clinically staged at the time of diagnosis using the 7th edition of the American Joint Committee on Cancer (AJCC) [18]. Clinical stage (1 and 2 vs. 3 vs. 4), tumour receptor status (oestrogen receptor (ER) + /progesterone receptor (PR) + / human epidermal growth factor receptor (HER2)- vs. ER + /PR + /HER2 + vs ER-/PR-/HER2 + vs ER-/PR-/HER2-, and treatment information (surgery, chemotherapy, and radiation therapy) were collected directly from the medical record.

We collected data on seven chronic conditions: hypertension, obesity, diabetes, HIV, cerebrovascular disease, asthma/chronic obstructive pulmonary disease (COPD), and tuberculosis. The women were asked if they had ever been treated for tuberculosis or ever been diagnosed and treated for hypertension, diabetes, cerebrovascular disease, and asthma/COPD. Obesity (body mass index (BMI) ≥ 30.0 kg/m^2^) was calculated from body weight and height measurements taken at enrolment; HIV status was tested for consenting patients through the National Health Laboratory Services, using an enzyme-linked immunosorbent assay. We defined multimorbidity as having ≥ 2 of these seven chronic conditions.

### Outcome data

Our primary outcome was overall survival. Patients were contacted by telephone every 3 months to determine their vital status. If the patient, next of kin, and other person named as close contacts were unable to be reached for 2 consecutive follow-up calls, we searched the publicly available administrative database to determine the patient’s vital status. Sources of the date of death information were 74.2% from next of kin, 5.3% from hospital records, and 20.5% from publicly available administrative data. The cause of death data in this cohort was from multiple sources and may not be reliable enough to enable us to compute the exact proportions of excess deaths attributable to breast cancer and those due to complications of multimorbidity. Patients were censored at the last date when they were known to be alive if no additional information about vital status could be obtained.

### Statistical analysis

The socio-demographic, lifestyle, and clinical factors associated with each chronic condition and multimorbidity (≥ 2 of these chronic conditions not including breast cancer) were described using Pearson's chi-squared and Fisher's exact tests. Survival analyses were conducted on a time-since diagnosis scale, with the at-risk period commencing on the date of histologically confirmed breast cancer diagnosis and ending on the earliest date of death from any cause (i.e. mortality ‘failure’ of interest), the date on which the participant was last known to be alive [19], or administrative censoring date on August 31, 2021. We constructed Kaplan–Meier survival curves stratified by multimorbidity for the full cohort and stratified by age and stage at diagnosis. Survival comparisons were performed using the log-rank test.

In Cox proportional hazards models, we examined each chronic condition, combinations of chronic conditions, and multimorbidity as separate binary indicators of all-cause mortality, without adjustment in model 1, adjusting for age in model 2, and in model 3 we further adjusted for known survival determinants which were possible confounders of the unadjusted association: i.e. age categories, the highest level of education, household socio-economic status, stage, receptor status, and hospital site. All adjusted variables were categorical with age group as a 10-year band from > 40 years. All statistical analyses were performed using Stata version 16 (StataCorp Ltd., College Station, TX).

## Results

A total of 3,497 women were enrolled in the SABCHO Study between July 2015 and December 2019. After excluding patients with missing information on hypertension, diabetes, cerebrovascular disease, asthma/COPD, tuberculosis, height, weight, and HIV status, 3,261 women were included in the final analyses. The most prevalent chronic conditions were obesity (53%), hypertension (41%), HIV (22%), and diabetes (13%), whereas asthma/COPD had the lowest prevalence (4%) (Table 1). The prevalence of these chronic conditions was higher in older women (≥ 50 years of age), except for HIV where 67.5% of women were < 50 years old. Most women in the cohort were unmarried and educated. Smoking prevalence though low overall ranged from 8% in HIV-positive women to 23% in those with asthma/COPD (Table 1).

**Table 1:** Socio-demographic and lifestyle characteristics of women with breast cancer in the SABCHO Study by chronic conditions

	Chronic conditions (*N* = 3261)							
	None	Obesity	Hypertension	Diabetes	Cerebrovascular disease	Asthma/COPD^b^	HIV	Tuberculosis
Number with chronic conditions (%)	569 (17.4)	1741 (53.4)	1329 (40.8)	419 (12.9)	195 (6.0)	144 (4.4)	727 (22.3)	215 (6.6)
Number without chronic condition (%)	2692 (72.6)	1,520 (46.6)	1,932 (59.2)	2,842 (87.1)	3,066 (94.0)	3,117 (95.6)	2,534 (77.7)	3,046 (93.4)
Characteristics among those with chronic condition	*N* = 569	*N* = 1741	*N* = 1329	*N* = 419	*N* = 195	*N* = 144	*N* = 727	*N* = 215
Age in years, mean ± SD	52.7 ± 15.3	55.7 ± 13.0	63.7 ± 11.7	64.2 ± 10.8	64.2 ± 11.6	57.3 ± 11.3	46.2 ± 10.0	51.8 ± 12.4
Age in years (%)								
< 50	281 (49.4)	619 (35.6)	154 (11.6)	40 (9.6)	23 (11.8)	41 (28.5)	491 (67.5)	105 (48.8)
≥ 50	288 (50.6)	1122 (64.4)	1175 (88.4)	379 (90.4)	172 (88.2)	103 (71.5)	236 (32.5)	110 (51.2)
Ethnicity (%)								
Asian	74 (13.0)	135 (7.8)	195 (14.7)	117 (27.9)	59 (30.3)	31 (21.5)	2 (0.3)	7 (3.3)
Black	392 (68.9)	1,446 (83.1)	988 (74.3)	262 (62.5)	100 (51.3)	88 (61.1)	711 (97.8)	200 (93.0)
Mixed	36 (6.3)	69 (4.0)	67 (5.0)	24 (5.7)	12 (6.2)	6 (4.2)	13 (1.8)	5 (2.3)
White	67 (11.8)	91 (5.2)	79 (5.9)	16 (3.8)	24 (12.3)	19 (13.2)	1 (0.1)	3 (1.4)
Marital status (%)								
Not married	331 (58.2)	1,028 (59.0)	894 (67.3)	282 (67.3)	131 (67.2)	96 (66.7)	481 (66.2)	147 (68.4)
Married/Cohabiting	238 (41.8)	713 (41.0)	435 (32.7)	137 (32.7)	64 (32.8)	48 (33.3)	246 (33.8)	68 (31.6)
Highest level of education (%)								
Primary education and below	141 (24.9)	506 (29.2)	555 (42.0)	171 (41.5)	86 (44.6)	48 (33.6)	159 (22.0)	70 (32.9)
Secondary education and above	426 (75.1)	1,229 (70.8)	765 (58.0)	241 (58.5)	107 (55.4)	95 (66.4)	565 (78.0)	143 (67.1)
Household socio-economic status (HSES) (%)								
0–5 (Low to medium)	429 (75.4)	1,299 (74.6)	984 (74.0)	287 (68.5)	132 (67.7)	101 (70.1)	649 (89.3)	179 (83.3)
6–7 (High)	140 (24.6)	442 (25.4)	345 (26.0)	132 (31.5)	63 (32.3)	43 (29.9)	78 (10.7)	36 (16.7)
^a^Hospital (%)								
CHBAH	200 (35.1)	681 (39.1)	480 (36.1)	118 (28.2)	55 (28.2)	34 (23.6)	305 (42.0)	67 (31.2)
CMJAH	171 (30.1)	427 (24.5)	276 (20.8)	66 (15.8)	28 (14.4)	37 (25.7)	152 (20.9)	30 (14.0)
Durban	100 (17.6)	283 (16.3)	281 (21.1)	136 (32.5)	70 (35.9)	43 (29.9)	88 (12.1)	51 (23.7)
Greys	90 (15.8)	311 (17.9)	270 (20.3)	93 (22.2)	39 (20.0)	24 (16.7)	151 (20.8)	60 (27.9)
Ngwelezana	8 (1.4)	39 (2.2)	22 (1.7)	6 (1.4)	3 (1.5)	6 (4.2)	31 (4.3)	7 (3.3)
Have you ever smoked? (%)								
No	455 (80.0)	1,582 (90.9)	1,161 (87.4)	356 (85.0)	152 (77.9)	111 (77.1)	666 (91.6)	190 (88.4)
Yes	114 (20.0)	159 (9.1)	168 (12.6)	63 (15.0)	43 (22.1)	33 (22.9)	61 (8.4)	25 (11.6)
Have you ever consumed alcohol? (%)								
No	440 (77.3)	1,444 (82.9)	1,096 (82.5)	367 (87.6)	162 (83.1)	118 (81.9)	557 (76.6)	163 (75.8)
Yes	129 (22.7)	297 (17.1)	233 (17.5)	52 (12.4)	33 (16.9)	26 (18.1)	170 (23.4)	52 (24.2)
Number of chronic conditions								
1		617 (35.4)	265 (19.9)	31 (7.4)	11 (5.6)	17 (11.8)	265 (36.5)	21 (9.8)
2		695 (39.9)	610 (45.9)	123 (29.4)	48 (24.6)	43 (29.9)	297 (40.8)	92 (42.8)
≥ 3		429 (24.7)	454 (34.2)	265 (63.2)	136 (69.8)	84 (58.3)	165 (22.7)	102 (47.4

^a^CHBAH (Chris Hani Baragwanath Academic Hospital), CMJAH (Charlotte Maxeke Johannesburg Academic Hospital)

^b^COPD (Chronic obstructive pulmonary disease). Missing data for the whole cohort: Highest level of education (*n* = 16)

**Fig. 1:**
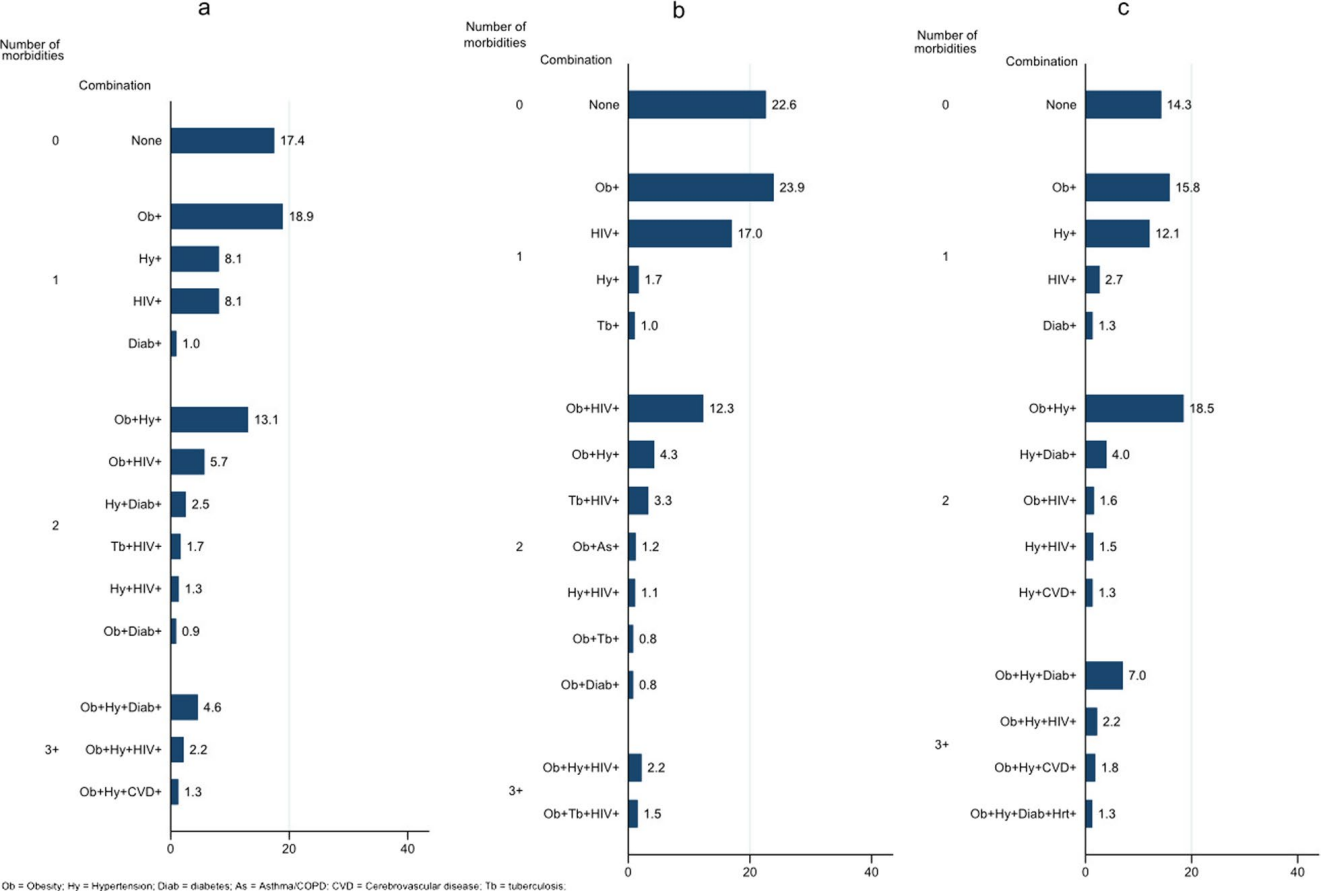
Profile of the combination of chronic conditions among women in the SABCHO cohort. **a** In the whole cohort. **b** In women < 50 years of age. **c** In women ≥ 50 years of age. Presented from highest to lowest percentage prevalence for each group

Only 17% of the women (14% of those ≥ 50 years and 23% of those < 50 years) had none of these seven chronic conditions (Fig. 1a–c). Forty-five per cent of the participants met our definition of multimorbidity; 29% of the women had only 2 chronic conditions and 16% had ≥ 3 chronic conditions. Among patients with multimorbidity, the most common combination was obesity and hypertension (13%), followed by obesity and HIV (6%) (Fig. 1a). Age ≥ 50 years, Asian ethnicity, unmarried status, and primary education or less were associated with having more chronic conditions. Most patients with 3 or more chronic conditions presented with stage I-II breast cancer (Table 2).

**Table 2:** Socio-demographic, lifestyle, and clinical characteristics of women with breast cancer in the SABCHO cohort by the number of chronic conditions at breast cancer diagnosis

	Multimorbidity				
	No	Yes		Total	
Number of chronic conditions	0–1	2	≥ 3		*p* value
Number of chronic conditions, N (row %)	*N* = 1796 (55.1)	*N* = 954 (29.2)	*N* = 511 (15.7)	*N* = 3261 (100.0)	
Age in years					< 0.001
< 50	835 (46.5)	314 (32.9)	93 (18.2)	1,242 (38.1)	
≥ 50	961 (53.5)	640 (67.1)	418 (81.8)	2,019 (61.9)	
Ethnicity					< 0.001
Asian	173 (9.6)	96 (10.1)	79 (15.5)	348 (10.7)	
Black	1,414 (78.7)	770 (80.7)	385 (75.3)	2,569 (78.8)	
Mixed	68 (3.8)	37 (3.9)	29 (5.7)	134 (4.1)	
White	141 (7.9)	51 (5.3)	18 (3.5)	210 (6.4)	
Marital status					< 0.001
Not married	1,056 (58.8)	607 (63.6)	348 (68.1)	2,011 (61.7)	
Married/Cohabiting	740 (41.2)	347 (36.4)	163 (31.9)	1,249 (38.3)	
Highest level of education					< 0.001
Primary education and below	464 (25.9)	307 (32.5)	206 (40.6)	977 (30.1)	
Secondary education and above	1,327 (74.1)	639 (67.5)	302 (59.4)	2,268 (69.9)	
Employment status					< 0.001
Unemployed	1,249 (69.5)	709 (74.3)	413 (80.8)	2,371 (72.7)	
Employed	547 (30.5)	245 (25.7)	98 (19.2)	890 (27.3)	
Household socio-economic status (HSES)					0.299
0–5 (Low to medium)	1,380 (76.8)	739 (77.5)	378 (74.0)	2,497 (76.6)	
6–7 (High)	416 (23.2)	215 (22.5)	133 (26.0)	764 (23.4)	
^a^ECOG					< 0.001
0–1	1,656 (93.5)	873 (92.8)	444 (87.9)	2,973 (92.4)	
2–4	115 (6.5)	68 (7.2)	61 (12.1)	244 (7.6)	
^b^Hospital					< 0.001
CHBAH	709 (39.5)	358 (37.5)	162 (31.7)	1,229 (37.7)	
CMJAH	482 (26.8)	218 (22.9)	85 (16.6)	785 (24.1)	
Durban	269 (15.0)	181 (19.0)	130 (25.4)	580 (17.8)	
Greys	299 (16.6)	172 (18.0)	123 (24.1)	594 (18.2)	
Ngwelezana	37 (2.1)	25 (2.6)	11 (2.2)	73 (2.2)	
Have you ever smoked?					0.187
No	1,565 (87.1)	850 (89.1)	440 (86.1)	2,855 (87.5)	
Yes	231 (12.9)	104 (10.9)	71 (13.9)	406 (12.5)	
Stage at diagnosis					0.044
Stages 1 and 2	728 (40.5)	388 (40.7)	237 (46.4)	1,353 (41.5)	
Stage 3	736 (41.0)	415 (43.5)	188 (36.8)	1,339 (41.1)	
Stage 4	332 (18.5)	151 (15.8)	86 (16.8)	569 (17.4)	
^c^Receptor subtype					0.030
ER + /PR + HER2-	1,085 (60.4)	595 (62.7)	300 (58.8)	1,975 (60.8)	
ER + /PR + HER2 +	307 (17.2)	153 (16.1)	76 (14.9)	536 (16.5)	
Er-&PR- HER2 +	134 (7.5)	52 (5.5)	30 (5.9)	216 (6.7)	
Er-&PR- HER2-	268 (15.0)	149 (15.7)	104 (20.4)	521 (16.0)	
Surgery					0.463
No	613 (34.1)	307 (32.2)	163 (31.9)	1,083 (33.2)	
Yes	1,183 (65.9)	647 (67.8)	348 (68.1)	2,178 (66.8)	
Chemotherapy					0.001
No	419 (23.3)	213 (22.3)	155 (30.3)	787 (24.1)	
Yes	1,377 (76.7)	741 (77.7)	356 (69.7)	2,474 (75.9)	
Radiation therapy					0.143
No	920 (51.2)	515 (54.0)	284 (55.6)	1,719 (52.7)	
Yes	876 (48.8)	439 (46.0)	227 (44.4)	1,542 (47.3)	

^a^ECOG (Eastern Cooperative Oncology Group)

^b^CHBAH (Chris Hani Baragwanath Academic Hospital), CMJAH (Charlotte Maxeke Johannesburg Academic Hospital)

^c^ER/PR (oestrogen receptor/progesterone receptor), HER2 (human epidermal growth factor receptor 2). Missing data: Highest level of education (*n* = 16), ECOG (*n* = 44), grade (*n* = 580), receptor subtype (*n* = 13)

**Fig. 2:**
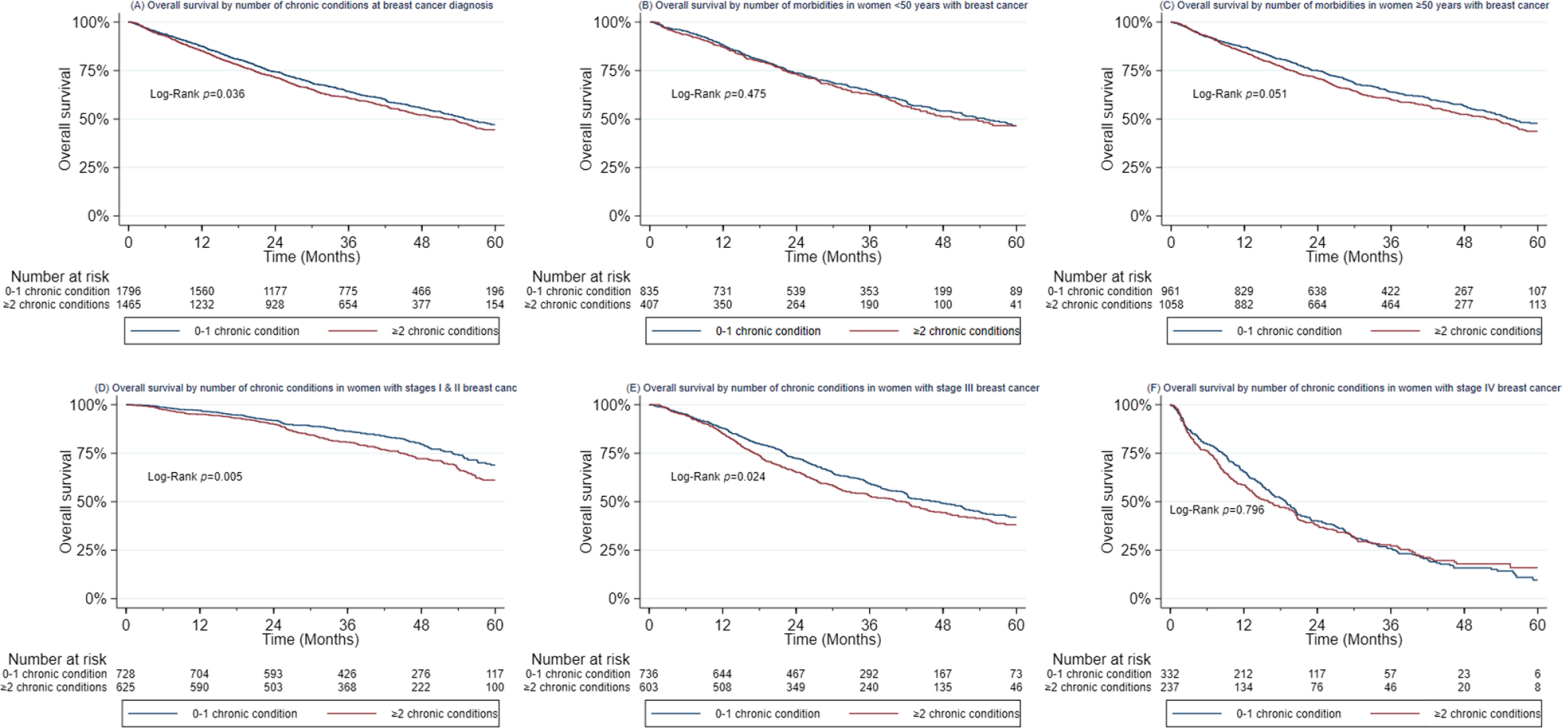
Breast cancer survival by the number of chronic conditions in the SABCHO cohort. Kaplan–Meier survival curves for mortality in women with breast cancer by the number of chronic conditions (0–1 vs ≥ 2) in the SABCHO cohort. **a** In the whole cohort. **b** In women < 50 years of age. **c** In women ≥ 50 years of age. **d** In women with stages I and II breast cancer. **e** In women with stage III breast cancer. **f** In women with stage IV breast cancer

With a median follow-up time of 32.6 months (IQR 19.5–48.8), 1410 (43.2%) of women had died. The median follow-up time was 32.7 months (20.0–49.0) with 743 (41.4%) deaths among those with < 2 chronic conditions, 32.1 months (18.4–48.6) with 667 (45.5%) deaths among those with ≥ 2 chronic conditions. The absolute survival at 2 years, 3 years, and 5 years is shown in Additional file 1: Table S1. Women with multimorbidity had poorer overall survival at 3 years than women without multimorbidity (60.8% vs. 64.3%, *p* = 0.036) in the full cohort, which was driven by a marginal survival difference in women ≥ 50 years old at breast cancer diagnosis (60.0% vs. 64.0%, *p* = 0.051), whereas there were no survival differences associated with multimorbidity among women < 50 years old at breast cancer diagnosis (*p* = 0.475) (Fig. 2). There was no age interaction with multimorbidity in the full cohort and in the analysis restricted to HIV-negative patients (data not shown). Multimorbidity was also associated with poorer 3-year survival among women with stage I and II disease (80.7% vs. 86.3%, *p* = 0.005), and stage III disease (53.0% vs. 59.4%, *p* = 0.024) but not among those with stage IV disease, all of whom had very low survival (27.1% vs. 25.9%, *p* = 0.796).

In model 1 (unadjusted), we found that women with obesity had lower all-cause mortality than women who were not obese (hazard ratio (HR) = 0.89, 95% confidence interval (CI) = 0.90–0.99), this HR attenuated and became non-significant in model 4. Women with CVD (HR = 1.30, 95% CI = 1.06–1.58), HIV (HR = 1.32, 95% CI = 1.17–1.49), and tuberculosis (HR = 1.23 95% CI = 1.01–1.50) had higher all-cause mortality than women without these conditions. Women who had obesity + HIV (HR = 1.20, 95% CI = 1.01–1.42), HIV + tuberculosis (HR = 1.39, 95% CI = 1.08–1.79), and multimorbidity (HR = 1.12, 95% CI = 1.01–1.24) also had higher mortality risks than women these conditions (Table 3). After adjustment for age, education, household socio-economic status, stage at diagnosis, receptor status, hospital site, and treatment received, the risks of mortality adjusted hazard ratios, given the conditions studied, were: diabetes (HR = 1.20, 95% CI = 1.03–1.41, *p* = 0.022), CVD (HR = 1.43, 95% CI = 1.17–1.76, *p* = 0.001), HIV (HR = 1.21, 95% CI = 1.06–1.38, *p* = 0.006), and obesity + HIV (HR = 1.24, 95% CI = 1.04–1.48, *p* = 0.017). Survival remained poorer for women with multimorbidity (≥ 2 chronic conditions) (HR = 1.26, 95% CI = 1.13–1.40, *p* < 0.001) than for women without multimorbidity (Table 3).

**Table 3:** Hazard ratios (95% CI) for all-cause mortality based on the presence of multimorbidity in women with breast cancer in the SABCHO cohort using Cox proportional hazards models

Chronic condition	Died	Model 1	Model 2	Model 3	Model 4	*p*-value for model 4
	*N* = 1410 (row %)	HR (95% CI)	HR (95% CI)	HR (95% CI)	HR (95% CI)	
Obesity	745 (41.6)	0.89 (0.80–0.99)^b^	0.92 (0.83–1.03)	0.96 (0.87–1.07)	1.05 (0.94–1.17)	0.392
Hypertension	575 (43.3)	0.99 (0.89–1.10)	0.95 (0.84–1.07)	1.04 (0.92–1.18)	1.05 (0.93–1.19)	0.409
Diabetes	201 (48.0)	1.12 (0.96–1.30)	1.11 (0.95–1.29)	1.21 (1.03–1.42)^a^	1.20 (1.03–1.41)^a^	0.022
Cerebrovascular disease	104 (53.3)	1.30 (1.06–1.58)^b^	1.27 (1.04–1.56)^a^	1.47 (1.19–1.80)^c^	1.43 (1.17–1.76)^b^	0.001
Asthma/COPD	69 (47.9)	1.04 (0.82–1.33)	1.09 (0.86–1.39)	1.17 (0.91–1.50)	1.22 (0.95–1.56)	0.119
HIV	353 (48.6)	1.32 (1.17–1.49)^c^	1.44 (1.26–1.64)^c^	1.32 (1.16–1.51)^c^	1.21 (1.06–1.38)^b^	0.006
Tuberculosis	110 (51.2)	1.23 (1.01–1.50)^b^	1.28 (1.05–1.56)^a^	1.16 (0.95–1.42)	1.16 (0.95–1.42)	0.137
Obesity + hypertension	336 (41.7)	0.94 (0.83–1.06)	0.94 (0.83–1.07)	1.04 (0.91–1.80)	1.09 (0.96–1.24)	0.177
Obesity + HIV	150 (47.9)	1.20 (1.01–1.42)^b^	1.28 (1.07–1.52)^b^	1.25 (1.05–1.50)^a^	1.24 (1.04–1.48)^a^	0.017
Hypertension + Diabetes	161 (48.1)	1.10 (0.94–1.31)	1.08 (0.91–1.28)	1.19 (1.00–1.41)	1.18 (1.00–1.41)	0.054
HIV + Tuberculosis	62 (53.9)	1.39 (1.08–1.79)^b^	1.48 (1.46–1.92)^b^	1.23 (0.95–1.60)	1.23 (0.95–1.60)	0.124
Multimorbidity (≥ 2 chronic conditions)	667 (45.5)	1.12 (1.01–1.24)^b^	1.15 (1.03–1.28)^a^	1.19 (1.07–1.32)^c^	1.26 (1.13–1.40)^c^	< 0.001

COPD (Chronic obstructive pulmonary disease). HR (Hazard ratio), CI (confidence interval)

Each chronic condition, combinations of chronic conditions and multimorbidity were added as a separate binary indicator to each model unadjusted in model 1, adjusting for age categories (< 40, 40–49, 50–59, 60–69, ≥ 70 years) in model 2, adjusted for age categories (< 40, 40–49, 50–59, 60–69, ≥ 70 years), education, household socio-economic status, stage, receptor status, and hospital site in model 3, and in model 4 we adjusted for age categories (< 40, 40–49, 50–59, 60–69, ≥ 70 years), education, household socio-economic status, stage, receptor status, hospital site, surgery, chemotherapy and radiation therapy

^a^(significant at *p* < 0.05)

^b^(significant at *p* < 0.01)

^c^(significant at *p* < 0.001)

To examine if the effect of the difference in survival by multimorbidity is mainly driven by HIV, we ran the model while excluding patients with HIV. We found that overall mortality based on multimorbidity status was marginally different in the whole cohort (*p* = 0.067) and in women ≥ 50 years (*p* = 0.072), but no difference in women < 50 years old (*p* = 0.912). By stage at diagnosis, women with multimorbidity still had worse survival in stage I and II disease (*p* = 0.006) and in stage III disease (*p* = 0.009), but not in stage IV disease (*p* = 0.831) than women without multimorbidity (Additional file 2: Supplementary Fig. S1).

## Discussion

In our cohort of 3261 women newly diagnosed with breast cancer, 44.7% of the women presented with multimorbidity (≥ 2 chronic conditions in addition to breast cancer). With a median follow-up time of 32.6 months, 43.2% of women had died. Women with obesity had better overall survival than non-obese women, but women living with diabetes, CVD, HIV, and obesity + HIV died earlier than women without these chronic conditions. Women with multimorbidity had higher mortality than women without multimorbidity in the whole cohort, a difference that was maintained when looking specifically at women with stages I and II, and those with stage III breast cancer but not in stage IV disease.

A large study in Spain found that breast cancer patients with a higher morbidity burden had poorer survival than those with no morbidity (HR: 2.33 95% CI: 1.76–3.08) [20]. These results support our findings and those of several other recent studies of survival given multimorbidity [15, 21]. Multimorbidity may adversely affect survival in several ways. Concomitant chronic disease may cause non-cancer mortality, as it would among patients without breast cancer [21–23]. Several studies have found that patients with multimorbidity receive less intensive treatment for their breast cancer than those without multimorbidity, potentially impacting their overall survival [12, 24]; from our findings, women with multimorbidity had a 26% higher mortality rate than women without multimorbidity when we adjusted for treatment received (Table 3, model 4) which was worse than the 19% higher mortality rate when we did not factor in treatment received (Table 3, model 3). If the chronic condition involves organ failure (*e.g.* compromised cardiac, respiratory, or renal function), curative treatment involving surgery, chemotherapy, and radiation therapy may not be possible, but undertreatment may then adversely affect their overall survival [25]. In addition, the chronic condition may make the patient more vulnerable to toxicity from chemotherapy and radiation therapy, leading to dose reduction or early discontinuation of treatment and, therefore, poorer outcomes [26, 27]. Also, patients with chronic conditions and multimorbidity have been found to have a higher risk of recurrence of their cancer than patients without additional conditions [21]. Clinicians caring for older women with a history of early-stage breast cancer may be concerned that concomitant chronic conditions will increase the toxicity and side effects of cancer treatment, that treatments may be less effective in these women, or that their life expectancy is insufficient to justify the use of potentially toxic agents [21, 28]. Such patients may also decline treatment for their cancer [29]. In addition, medications for the coexisting chronic conditions may affect the risk of side effects from cancer therapies mainly from drug-drug interaction and make treatment less effective [30], or a lack of focus on care for the coexisting chronic condition during cancer treatment may lead to poor survival [31]. Lastly, the risk of dying from a chronic condition may be so high that even patients who are diagnosed at an early stage and receive the recommended breast cancer treatment may not benefit from it [25]. Hence, there is a need for a study of causes of death generally in women with multimorbidity and breast cancer.

We found that women with stage IV breast cancer and multimorbidity did not have poorer overall survival than those without multimorbidity. This is consistent with the findings of other studies that multimorbidity has a greater impact on cancers with a better prognosis [32, 33]. Patients who have cancer associated with a high mortality rate are more likely to die from that cancer, regardless of their co-morbid diseases than patients who have a better cancer prognosis. Higher mortality rates in women with multimorbidity manifest 1 year post-diagnosis in stage III women and after 2 years in stages I and II women with breast cancer. The delayed effect of multimorbidity on lower survival might indicate that the lower survival is not due to higher background mortality which would apply to all periods, but due to a breast cancer-defined attribute, e.g. perhaps women with multimorbidity have lower treatment completion/quality and a thus great chance of recurrence and eventually lower survival.

In a report from the Shanghai Breast Cancer Survival Study of 4,664 women with stage I-III breast cancer, diabetes was associated with poorer overall survival [34] mainly from chronic vascular complications [13]. Other studies have reported poorer overall survival in women with breast cancer and CVD [13]. Our finding that HIV infection was associated with higher all-cause mortality is consistent with prior work from HICs demonstrating higher mortality rates among patients with HIV infection and breast cancer than among HIV-uninfected breast cancer patients [35, 36]. We too have published findings showing that the 2-year overall survival of women with stages I-III breast cancer was poorer among women living with HIV than among HIV-uninfected women as reported by Ayeni et al. (72.4% vs. 80.1%, *p* < 0.001) and adjusted hazard ratio (HR = 1.49, 95% CI = 1.22–1.83) [37]. Whilst we did not find any statistical evidence of an interaction with age, the *p* values were small (< 0.1); thus, in future larger studies it would be useful to reexamine whether the multimorbidity effect might be stronger at older ages.

The theory of syndemics offers an innovative way of understanding why diseases cluster together in populations disproportionately affected by poverty and other social and environmental stressors [8, 38]. A syndemic is the clustering of two or more diseases within a population that arise from and contribute to persistent economic and social inequalities [39]. Patients experience morbidity through a combination of complex social, environmental, and medical conditions that impact the quality of life and survival outcomes. The causes, courses, and outcomes of communicable diseases, nutritional conditions, and non-communicable diseases are closely related [40]. They all have common underlying social conditions such as unhealthy environments and poverty, and disease groups entail common comorbidities, causes, and care needs [41]. Individuals may have both communicable diseases and non-communicable diseases and one can increase the impact or risk of the other [42]. In South Africa, we need to recognize the different clusters of socio-economic status and multimorbidity in our women with breast cancer and how they interact with each other and affect health outcomes. This recognition could support new investigative approaches that integrate more than just disease-specific models, incorporating economic, environmental, and social conditions that exacerbate the effects of disease clusters. We must also incorporate syndemic considerations into policies for disease prevention and management of health systems. Screening for common comorbidities like obesity, hypertension, diabetes, HIV, depression, alcoholism, and algorithms for understanding and accommodating socio-economic circumstances for treatment plans should be routinely introduced for single clinic visits in primary care [8, 38, 39].

The strengths of our study include the prospective study design, multi-centre cohort, and large sample size. The 7 chronic conditions were included because of their known high prevalence in the least parts of South Africa where regular treatment may provide an avenue for early cancer detection (e.g. HIV and tuberculosis [43], COPD [44], hypertension and heart disease [45]), their known impact on treatment (e.g. cardiotoxicity and worsening of hypertension [46]), and their known association with breast cancer prognosis (obesity [47], diabetes [48], and HIV [49]). Among the limitations, the 236 participants excluded due to missing information on chronic conditions were not socio-demographically different from the final sample in the analyses; hence, our findings are generalizable. We note that we included only seven chronic conditions at baseline, we may therefore have underestimated the prevalence of chronic conditions and multimorbidity in this study. We also did not assess the severity of chronic conditions, and we did not investigate the role of newly diagnosed chronic conditions after the breast cancer diagnosis, because the objective of the present study was to investigate chronic conditions at diagnosis and survival outcomes. Five of the 7 chronic conditions were self-reported without additional objective laboratory testing and measurement of blood pressure at diagnosis, which may also have led us to underestimate the prevalence of chronic conditions in this cohort [50].

## Conclusion

This study confirmed that overall survival was significantly poorer among women with multimorbidity at breast cancer diagnosis than among other women without multimorbidity, particularly in women diagnosed with early-stage or locally advanced breast cancer. Future studies should assess and report the influence of multimorbidity and syndemics on breast cancer treatments and breast cancer-specific mortality.

## Supplementary Information

**Additional file 1: Table S1**. Absolute survival differences overall and by the number of chronic conditions in the SABCHO cohort.

**Additional file 2: Figure S1**. Kaplan–Meier survival curves for mortality in HIV-negative women with breast cancer by number of chronic conditions (A) Overall; (B) and (C) by age categories, and (D), (E), and (F) by stage at diagnosis in the SABCHO cohort.

## Data Availability

The statistical output data underlying this article will be shared upon reasonable request to the corresponding author.

## Abbreviations

BMI: Body mass index
CHBAH: Chris Hani Baragwanath Academic Hospital
CI: Confidence interval
CMJAH: Charlotte Maxeke Johannesburg Academic Hospital
COPD: Chronic Obstructive Pulmonary Disease
CVD: Cerebrovascular disease
HICs: High-income countries
HIV: Human immunodeficiency virus
HR: Hazard ratio
HSES: Household socio-economic status
IQR: Interquartile range
LMICs: Low- and middle-income countries
SABCHO: South African Breast Cancer and HIV Outcomes Study
