# Correction: Multimorbidity and overall survival among women with breast cancer: results from the South African Breast Cancer and HIV Outcomes Study

**DOI:** 10.1186/s13058-023-01611-w

**Published:** 2023-01-31

**Authors:** Oluwatosin A. Ayeni, Maureen Jofe, Witness Mapanga, Wenlong Carl Chen, Daniel S. O’Neil, Boitumelo Phakathi, Sarah Nietz, Ines Buccimazza, Sharon Čačala, Laura W. Stopforth, Judith S. Jacobson, Katherine D. Crew, Alfred I. Neugut, Duvern Ramiah, Paul Ruf, Herbert Cubasch, Tobias Chirwa, Valerie McCormack, Lisa K. Micklesfeld, Shane A. Norris

**Affiliations:** 1 Strengthening Oncology Services Research Unit, Faculty of Health Sciences, University of the Witwatersrand, Johannesburg, South Africa; 2 Department of Radiation Oncology, Faculty of Health Sciences, University of the Witwatersrand, Johannesburg, South Africa; 3 SAMRC/Wits Developmental Pathways for Health Research Unit, Department of Paediatrics, Faculty of Health Sciences, University of the Witwatersrand, Johannesburg, South Africa; 4 National Cancer Registry, National Health Laboratory Service, Johannesburg, South Africa; 5 Sydney Brenner Institute for Molecular Bioscience, Faculty of Health Sciences, University of the Witwatersrand, Johannesburg, South Africa; 6 Sylvester Comprehensive Cancer Center and Department of Medicine, Miller School of Medicine, University of Miami, Miami, FL USA; 7 Department of Surgery, School of Clinical Medicine, University of KwaZulu-Natal, KwaZulu-Natal, South Africa; 8 Department of Surgery, Faculty of Health Sciences, University of the Witwatersrand, Johannesburg, South Africa; 9 Department of Surgery, Ngwelezana Hospital, Empangeni and University of KwaZulu-Natal, Empangeni, KwaZulu-Natal South Africa; 10 Departments of Surgery and Radiation Oncology, Grey’s Hospital, University of KwaZulu-Natal, Pietermaritzburg, KwaZulu-Natal South Africa; 11 Herbert Irving Comprehensive Cancer Center, Vagelos College of Physicians and Surgeons, Columbia University, New York, NY USA; 12 Department of Epidemiology, Mailman School of Public Health, Columbia University, New York, NY USA; 13 Department of Medicine, Vagelos College of Physicians and Surgeons, Columbia University, New York, NY USA; 14 Division of Medical Oncology, Department of Internal Medicine, Faculty of Health Sciences, University of the Witwatersrand, Johannesburg, South Africa; 15 School of Public Health, Faculty of Health Sciences, University of the Witwatersrand, 27 St Andrews Road, Parktown, Johannesburg, 2193 South Africa; 16 Environment and Lifestyle Epidemiology Branch, International Agency for Research On Cancer, (IARC/WHO), Lyon, France; 17 Global Health Research Institute, School of Human Development and Health, University of Southampton, Southampton, UK

**Correction: Breast Cancer Res 25, 7 (2023)** https://doi.org/10.1186/s13058-023-01603-w

Following publication of the original article [1], the authors reported an error in the title. There is a break in the title and a large blank space besides the “background” section.

The original article [1] has been updated.
