# Supplementary material for: Multimorbidity and overall survival among women with breast cancer: results from the South African Breast Cancer and HIV Outcomes Study
Source: Breast Cancer Res. 2023 Jan 23;25:7. doi: 10.1186/s13058-023-01603-w (PMC9872426; doi:10.1186/s13058-023-01603-w)
Supplement: Supplementary file 1 — Additional file 1: Table S1. Absolute survival differences overall and by the number of chronic conditions in the SABCHO cohort. [file 13058_2023_1603_MOESM1_ESM.pdf]

Supplementary Table 1 Absolute survival differences overall and by the number of chronic conditions in the SABCHO cohort.

**Absolute survival**	**Total (All)**	**<2 chronic conditions**	**≥2 chronic conditions**
	**% (95%CI)**	**% (95%CI)**	**% (95%CI)**
**2 years**	73.1% (71.5 – 74.6)	74.4% (72.3 – 76.4)	71.5% (69.1 – 73.8)
**3 years**	62.7% (60.9 – 64.4)	64.3% (61.9 – 66.6)	60.8% (58.1 – 63.3)
**5 years**	45.9% (43.6 – 48.2)	47.1% (44.0 – 50.2)	44.4% (41.1 – 47.7)

% (Percentage surviving), CI (Confidence interval)
