# Supplementary figures and images for: Multimorbidity and overall survival among women with breast cancer: results from the South African Breast Cancer and HIV Outcomes Study
Source: Breast Cancer Res. 2023 Jan 23;25:7. doi: 10.1186/s13058-023-01603-w (PMC9872426; doi:10.1186/s13058-023-01603-w)

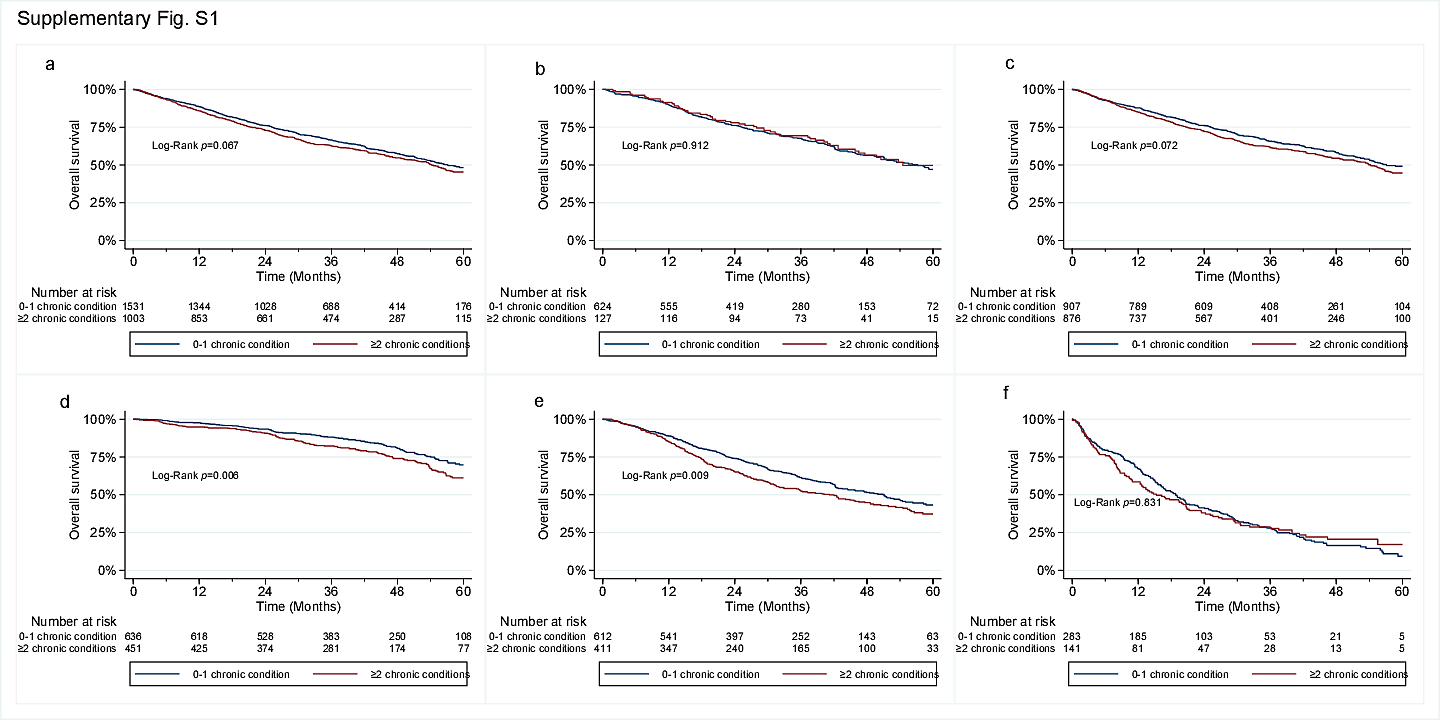

Supplement: Supplementary file 2 — Additional file 2: Figure S1. Kaplan–Meier survival curves for mortality in HIV-negative women with breast cancer by number of chronic conditions (A) Overall; (B) and (C) by age categories, and (D), (E), and (F) by stage at diagnosis in the SABCHO cohort. [file 13058_2023_1603_MOESM2_ESM.tiff]
